# Incidence and nature of functional voice disorders

**DOI:** 10.4102/sajcd.v72i1.1117

**Published:** 2025-11-25

**Authors:** Yusra M. Bhaila, Jeannie van der Linde, Carmen Milton, Marien A. Graham

**Affiliations:** 1 Department of Speech Therapy and Audiology, Faculty of Humanities, University of Pretoria, Pretoria, South Africa; 2 Department of Early Childhood Education, Faculty of Education, University of Pretoria, Pretoria, South Africa; 3 Department of Mathematics Education, College of Education, University of South Africa, Pretoria, South Africa

**Keywords:** functional voice disorders, speech-language pathology, voice disorders, occupational voice users, laryngeal pharyngeal reflux, muscle tension dysphonia

## Abstract

**Background:**

Functional voice disorders (FVDs) result from inefficient vocal use without an organic cause and have an underreported incidence.

**Objectives:**

This study aimed to investigate the incidence and nature of FVDs among treatment-seeking patients at a private interdisciplinary voice clinic in Pretoria, South Africa.

**Method:**

A retrospective quantitative design was used to analyse data from 86 patients diagnosed with FVDs between January 2017 and July 2022. Descriptive and inferential statistics were applied.

**Results:**

The incidence rate of FVDs was 16.67% among 516 patients with voice disorders. The primary diagnosis was muscle tension dysphonia (MTD), affecting 80.2% of cases. The most common secondary diagnosis was laryngeal pharyngeal reflux (LPR) at 39.5%. Hoarseness was the most reported symptom. No significant differences were found between occupational and non-occupational voice users.

**Conclusion:**

The findings clarify the incidence and nature of FVDs in the South African context.

**Contribution:**

It underscores the need for greater awareness of FVDs in low- and middle-income countries (LMICs) as well as future research on prevention and management strategies.

## Introduction

Functional voice disorders (FVDs) stem from unhygienic use of the vocal mechanism, with the absence of anatomical or neurological abnormalities (Naqvi & Gupta, 2021). Voice disorders have a prevalence rate of 7.5% in the United States of America (US) (Cohen et al., 2017); yet specific incidence rates as well as the nature of FVDs have been inadequately reported globally.

Functional voice disorders can include, but are not limited to, vocal fatigue, muscle tension dysphonia (MTD), diplophonia, ventricular phonation and psychogenic voice disorders (Naqvi & Gupta, 2022). Functional voice disorders are known to reduce job performance and, ultimately, quality of life (Behlau et al., 2012). As such, vocal quality, pitch and loudness can be impaired without any contributing anatomical or neurological impairments (Chung et al., 2018). Given the varying nature of FVDs, treatment will differ (Naqvi & Gupta, 2021). Thus, describing the incidence and nature of FVDs will guide professionals to render appropriate services from the onset.

A substantial proportion of the labour force consists of professionals who rely on their voice, also known as occupational voice users (OVUs) (Roy et al., 2016). An OVU is defined as an individual who requires their voice to complete their day-to-day work activities (Phyland & Miles, 2019). This can include, but is not limited to, singers, stage performers, sports coaches, sales assistants, teachers, lecturers, lawyers, telephone operators, call centre workers, receptionists, priests and health professionals (Phyland & Miles, 2019). More longitudinal studies are needed to follow the development and treatment of FVDs (Cantor Cutiva et al., 2013). Most OVUs are at an increased risk of developing an FVD (Phyland & Miles, 2019). This has been illustrated in the literature among the teaching (Cantor Cutiva et al., 2013), singing (Ravall & Simberg, 2020) and physician populations (El Tayeb & Abdo, 2012).

Functional voice disorders among the OVU population limit a professional’s working capacity and have health implications that require medical, speech and psychological intervention (Al-Saleem & Alsaleem, 2013). Management of voice disorders is a costly public health challenge, because of medical treatment and its impact on the workforce and employment (Cohen et al., 2017). In the US, voice disorders are estimated to cost $5 billion annually, combining direct healthcare costs and indirect costs related to work absenteeism and presenteeism (Cohen et al., 2017). Consultations with general medical practitioners (GMPs) appear to be counterproductive for voice problems as they do not have sufficient knowledge of voice disorders to ensure adequate intervention (Cohen et al., 2017). Therefore, patients with FVDs do not receive an otorhinolaryngology evaluation upon entry, and this often results in inaccurate diagnoses and inappropriate treatment, which adds to treatment costs. Epidemiological data have a direct effect on service delivery as they enable an earlier and correct diagnosis (Cohen et al., 2017). This also enables rapid and appropriate service provision while decreasing costs (Cohen et al., 2017).

Research carried out in Saudi Arabia found that 80.9% of male teachers from a sample size of 340 teachers had experienced voice-related problems in the previous year (Al-Saleem & Alsaleem, 2013). A systematic review conducted in the Netherlands reported a point prevalence of 9% – 37% and a 12-month prevalence of 15% – 80% of voice disorders among teachers in both low- and middle-income countries (LMICs) and high-income countries (HICs); of which three publications included a clinically verified prevalence of 17% – 57% and seven studies reported a lifetime prevalence of 51% – 69% (Cantor Cutiva et al., 2013). Within the teaching population, both HICs (including Saudi Arabia, the Netherlands, the United States, Finland and Latvia) and LMICs (such as Iran) produced similar results regarding prevalence. Thus, the incidence of FVDs has been inadequately recorded globally.

Epidemiological studies aimed at determining the frequency of voice disorders in several populations in Brazil, Iran, Latvia, Finland and Egypt. Teachers in Brazil (Behlau et al., 2012), Iran (Seifpanahi et al., 2016) and Latvia experienced a voice disorder in their lifetime (Trinite, 2017). A study conducted in Finland investigated the association between choir singing and voice disorders, and 21% presented with an FVD because of their vocally harmful behaviour (Ravall & Simberg, 2020). Similarly, the prevalence of FVDs in Egypt was significantly higher among private physicians when compared to lab physicians (El Tayeb & Abdo, 2012). It should be observed that despite the geographical differences and the variant sample sizes, the research findings were similar among the different populations.

Most studies, which investigated the incidence and/or prevalence of FVDs, were conducted and published in HICs, particularly in North America and Europe. Studies carried out in Asia were conducted in Saudi Arabia and Iran, while one study in Africa was carried out in Egypt. It is important to observe that other continents, including South America and Australia, did not yield any literature pertaining to the incidence of FVDs. Studies exploring the incidence and prevalence of FVDs are evidently needed in LMICs because of the limited published research available.

To date, there is no research available on FVDs in South Africa (SA). Therefore, there is a need to pursue research in this area as it is a global health challenge. Information on the nature of FVDs that are diagnosed and managed within the South African population will advocate for optimum management including prevention and rehabilitation services (Tenny & Boktor, 2022). The high incidence and prevalence of FVDs, especially in OVUs, substantiate the need for prevention strategies, early identification and treatment of FVDs (Fourie et al., 2017). In addition, data on the incidence of FVDs may inform the aetiology of the condition, its associated risk factors and its potential outcomes. In turn, these statistics advocate for the allocation of medical resources through efficient planning of service delivery within the interdisciplinary teams consisting of ear, nose and throat (ENT) specialists and speech-language pathologists (SLPs), specifically in LMICs (Cohen et al., 2014).

There is a pressing need for data on FVDs both within the South African context and internationally, owing to limited published research from LMICs. Identifying the incidence of FVDs will provide a better understanding of the nature of the disorder and may inform and improve prevention strategies, address risk factors and improve clinical outcomes. Based on the aforementioned, the following research question is posed: What is the incidence and nature of FVDs in adults at a South African interdisciplinary voice clinic?

## Research methods and design

The study aimed to describe the incidence and nature of FVDs in adults at an interdisciplinary voice clinic in SA. A retrospective quantitative research design was utilised. Institutional Review Board (IRB) clearance was obtained from the Faculty of Humanities and the Faculty of Health Sciences (Reference number: HUM003/0822) prior to data extraction and data analysis.

Data were extracted from an interdisciplinary Voice Clinic’s secure medical database, where patient-related healthcare information is stored as part of standard patient healthcare records. The data were captured and anonymised during data extraction to ensure confidentiality of personal information was upheld. Male and female participants, over the age of 18 years with a confirmed diagnosis of an FVD, who attended the Voice Clinic between January 2017 and July 2022 were included in the dataset (refer to Table 1).

**TABLE 1:** Data categories and justification for their inclusion.

Categories	Justification
Number of participants diagnosed with an FVD (Incidence)	There is currently no incidence data in South Africa on FVDs.
Date of diagnosis	The date of diagnosis along with the treatment and outcomes will contribute to understanding the nature of FVDs.
Age	The prevalence of voice disorders increases with age (65 years and older) (Bainbridge et al., 2017). Young and middle-aged adults are least likely to experience a voice disorder (Bainbridge et al., 2017).
Gender	Women are likely to experience vocal health issues more than men, regardless of occupation. This is because of the difference in the laryngeal structure (Hunter et al., 2011). Furthermore, in a study conducted by Bainbridge et al. (2017), females had a 56% higher chance of experiencing a voice disorder than males. Thus, it is essential to understand gender distinctions and gender-specific characteristics.
Referring physician	To highlight the number of patients referred for an otolaryngology evaluation from different healthcare workers, ranging from general medical practitioners to specialist physicians. In addition, it will allow for quantifying of the number of self-referrals from the given population.
Hydration	Inadequate systematic hydration can result in vocal dryness and a hoarse voice (Alves et al., 2019). In addition, patients with functional dysphonia who received adequate hydration presented with an improved laryngeal state (Alves et al., 2019).
Allergies	A study conducted among a sample of patients with FVDs found that 76.32% of patients with dysphonia had co-occurring allergies (Lauriello et al., 2011). Moreover, a statistically significant association was found between the female gender and the presence of an allergy (Lauriello et al., 2011).
Smoking	Smoking has a significantly negative impact on the voice and can impact on MPT, VHI and acoustic factors (Byeon & Cha, 2020).
Alcohol consumption	A positive correlation between alcohol consumption and voice disorders was found in a study performed by Bainbridge et al. (2017).
Exercise	High levels of physical activity can result in an elevated fundamental frequency and can impact on vocal stability (Primov-Fever et al., 2013). This stems from an increase in vocal effort and can result in vocal pathology (Primov-Fever et al., 2013).
Occupation	Voice disorders are frequent among the OVU population (Brisson et al., 2024). Thus, it would be necessary to determine if patients are OVUs as this is a risk factor for acquiring voice disorder (Brisson et al., 2024).
Signs and symptoms	The most common symptoms of an FVD resulting from inefficient use of the vocal mechanism include MTD, aphonia, diplophonia and ventricular phonation (Naqvi & Gupta, 2022).
RSI	There are associations between subjective reports of symptoms of laryngopharyngeal reflux and voice disorders (Alanazi et al., 2018).
VHI	The VHI is a useful tool to quantify the biopsychosocial impact that a voice disorder has on an individual. Moreover, it will provide the researcher with insight into the nature of FVDs.
ENT diagnosis or diagnoses	The study focuses primarily on the diagnosis of FVDs as described by Naqvi and Gupta (2022).
SLP findings: Perceptual and acoustic findings	The SLP findings should match the general description of a FVD, along with the ENT assessment conclusions.
Referral from ENT to SLP	Voice therapy is mandated for patients with an FVD (Naqvi & Gupta, 2022). Healthcare services are optimised by providing long-term patient-centred care using an interdisciplinary approach (Naqvi & Gupta, 2022). Thus, services can be improved by ensuring that appropriate referrals are made to the SLP timeously.

Note: The data categories and justification framework were developed by the authors. The clinical data populating these categories were obtained from Dr Carl Swanepoel and Giselle Maartens of the Pretoria Voice Clinic, with permission. Please see the full reference list of the article, Bhaila, Y.M., Van der Linde, J., Milton, C., & Graham, M.A. (2025). Incidence and nature of functional voice disorders. *South African Journal of Communication Disorders, 72*(1), a1117. https://doi.org/10.4102/sajcd.v72i1.1117, for more information.

FVD, functional voice disorder; MPT, maximum phonation time; VHI, voice handicap index; OVU, occupational voice user; ENT, ear, nose, and throat; MTD, muscle tension dysphonia; RSI, reflux symptom index; VHI, Voice Handicap Index; SLP, speech-language pathologists.

The data were analysed using the Statistic Package of Social Sciences (SPSS) for Windows, Version 28.0 (IBM Corp., Armonk, NY, United States), which analysed the data by generating descriptive statistics (frequencies and percentages) to summarise the variables and applying inferential tests to examine differences between groups. The extracted data were used to determine the incidence of FVDs among the treatment-seeking population and the nature of the FVD.

Lifestyle and health factors were also explored, and this included hydration, smoking, alcohol consumption, allergies and exercise. The voice handicap index (VHI) was chosen as it is a validated and widely used tool that allows reliable comparison across groups (Caffier et al., 2021).

Descriptive and inferential statistics were used. In the analysis, the Mann–Whitney test was employed to assess potential differences between non-occupational and OVUs. The statistic was utilised for comparisons and for interpretation to understand potential significant differences between OVUs and non-OVUs in relation to *p*-values.

### Ethical considerations

Ethical approval to conduct this study was obtained from the University of Pretoria’s research Ethics Committee (reference number: HUM003/0822).

## Results

Out of the total number of patients who attended the voice clinic (*n* = 516) between January 2017 and July 2022, 86 (16.67%) patients were diagnosed with an FVD. The data from the 86 patients with FVDs were analysed for this study. From the sample, 24.4% (*n* = 21) were male patients and 75.6% (*n* = 65) were female patients. Their ages ranged from 18 years to 77 years old (mean = 47.26; standard deviation [s.d.] = 13.643). Many patients who presented with a primary diagnosis of an FVD (100%) presented with a secondary ENT-related diagnosis (58.1%; *n* = 50). The most prevalent primary diagnoses of FVDs were MTD (80.2%; *n* = 69) followed by functional dysphonia (12.8%; *n* =11).

Naqvi and Gupta (2022) describe MTD as one of the most common types of FVDs, although the terms MTD, functional dysphonia and hyper-functional dysphonia are also used interchangeably. Muscle tension dysphonia causes hypertonicity of the laryngeal musculature, which restricts the vocal folds’ capacity to abduct and adduct with co-ordination and the required speed (Naqvi & Gupta, 2022). Functional dysphonia occurs when the extrinsic and intrinsic laryngeal muscles are dysregulated (Roy, 2003).

Furthermore, 39.5% (*n* = 34) of patients presented with laryngeal pharyngeal reflux (LPR) as a secondary diagnosis; while 26.41% (*n* = 23) had abnormal RSI scores (mean = 23.01; s.d. = 14.365). Most of the patients (*n* = 65; 75.6%) scored high on the VHI (mean = 67.00; s.d. = 34.710). Muscle tension dysphonia may appear as both a primary and secondary diagnosis, as recorded by the ENT in the dataset. Table 2 summarises the diagnoses.

**TABLE 2:** Primary and secondary diagnoses.

Diagnoses	*n*	%
**Primary diagnosis (*N* = 86)**		
Functional dysphonia	11	12.8
Hyper-functional dysphonia	4	4.7
Muscle tension dysphonia	69	80.2
Paradoxical vocal fold movement disorder	2	2.3
**Secondary diagnoses (*N* = 50)**		
Acid reflux	1	1.2
Functional dysphonia with laryngospasms	1	1.2
Laryngeal candidiasis	1	1.2
Laryngeal pharyngeal reflux	34	39.5
Laryngospasm	1	1.2
Muscle tension dysphonia	4	4.6
Mutational falsetto	1	1.2
Myasthenia gravis	1	1.2
Non-specific laryngitis	1	1.2
Paradoxical vocal fold movement disorder	3	3.5
Septal deviation with spur	1	1.2
Upper respiratory inflammation	1	1.2

Note: Raw data obtained from Dr Carl Swanepoel and Giselle Maartens, Pretoria Voice Clinic. Table developed and calculations performed by the authors.

Lifestyle and health factors that were explored included hydration, smoking, alcohol consumption, allergies and exercise. The results are reflected in Table 3. While 18.6% (*n* = 16) of the participants had inadequate hydration (i.e. consumed less than two litres of water a day), 3.5% (*n* = 3) exercised moderately. Most of the participants (73.3%, *n* = 63) were non-smokers and 8.2% (*n* = 7) were smokers, while 41.9% (*n* = 36) did not consume alcohol. The majority of the participants (75.6%; *n* = 65) did not have any allergies.

**TABLE 3:** Lifestyle and health factors.

Participants’ characteristics	*n*	%
Hydration: Inadequate	16	18.6
Hydration: Adequate	33	38.4
Smoking: Non-smokers	63	73.3
Smoking: Smokers	7	8.2
Alcohol consumption: Non-drinkers	36	41.9
Alcohol consumption: Drinkers	29	33.7
Allergies: None	65	75.6
Exercise: Moderate	3	3.5

Note: Raw data obtained from Dr Carl Swanepoel and Giselle Maartens, Pretoria Voice Clinic. Table developed and calculations performed by the authors.

The perceptual assessment of voice quality of 65 participants (75.58%) was analysed using the Grade, Roughness, Breathiness, Asthenia, Strain (GRBASI) scale.

The mean for each of the GRBASI scores ranged between 0 and 3 for each component, namely grade; roughness; breathiness; asthenia; strain; instability; where 0 = normal; 1 = slight, 2 = moderate, 3 = severe. Among the participants, 26 (30.2%) had a severe grade score, 26 (30.2%) had a slight roughness score, 24 (27.9%) had a normal breathiness score, 24 (27.9%) had a normal asthenia score, 19 (22.1%) had a normal strain score and 25 (29.1%) had a normal instability score. The results can be viewed in Table 4.

**TABLE 4:** A summary of grade, roughness, breathiness, asthenia, strain and instability scores.

Parameter	Grade (*N* = 66)		Roughness (*N* = 65)		Breathiness (*N* = 65)		Asthenia (*N* = 64)		Strain (*N* = 65)		Instability (*N* = 64)	
	*n*	%	*n*	%	*n*	%	*n*	%	*n*	%	*n*	%
0	9	10.5	23	26.7	24	27.9	24	27.9	19	22.1	25	29.1
1	22	25.6	26	30.2	13	15.1	18	20.9	15	17.4	15	17.4
2	9	10.5	12	14.0	14	16.3	11	12.8	20	23.3	19	22.1
3	26	30.2	4	4.7	14	16.3	11	12.8	11	12.8	5	5.8

Note: Raw data obtained from Dr Carl Swanepoel and Giselle Maartens, Pretoria Voice Clinic. Table developed and calculations performed by the authors.

Most participants reported multiple signs and symptoms relating to the function of their voice. The most common symptoms reported were hoarseness (*n* = 58; 67.4%), throat irritation (*n* = 23; 26.7%), aphonia (*n* = 17; 19.8%), globus (*n* = 19; 22.1%), vocal fatigue (*n* = 18; 20.9%), difficulty with projection (*n* = 17; 19.8%) and increased throat clearing (*n* = 19; 22.1%). Other symptoms included: high-pitched voice (*n* = 6; 7.0%), low pitched voice (*n* = 3; 3.5%), strained voice (*n* = 13; 15.1%), loss of breath support (*n* = 9; 10.5%), chronic cough (*n* = 4; 4.7%), poor intelligibility (*n* = 1; 1.2%), painful voice (*n* = 5; 5.8%) and weak voice (*n* = 11; 12.8%). Psychological symptoms relating to depression and anxiety were also reported (*n* = 5; 5.8%).

The reported symptoms were analysed according to the number of patients from the sample who experienced one, two or more symptoms. The distribution of reported symptoms among the participants showed that for two patients (2.3%), no symptoms were reported, while 26 (30.2%) reported one symptom and 15 (17.4%) reported two or three symptoms. In addition, 12 patients (14.0%) reported four symptoms, and 9 individuals (10.5%) reported five symptoms. Only 3 individuals reported six or seven symptoms (3.5%) in each category, and 1 individual (1.2%) reported experiencing eight symptoms.

The two groups namely, OVUs versus non-OVUs, categorised according to the use of their voice as indispensable for their work or not, were compared (Phyland & Miles, 2019). There were 31 patients out of the sample population who were OVUs (43.66%; mean = 3.32) and 40 non-OVUs (56.35%; mean = 2.73). According to the results of the Mann–Whitney test, no significant difference between the two groups was observed (*p*-value = 0.247, *z* score = 1.156, MW score = 522.000).

Acoustic measures were also analysed. All the correlations between the age of patient, reflux symptom index (RSI), VHI, jitter, shimmer, fundamental frequency, noise-to-harmonic ratio and maximum phonation time were not significantly different as all *p*-values were > 0.05. The results can be viewed in Table 5.

**TABLE 5:** Comparison between occupational and non-occupational voice users.

Participants’ characteristics	Acoustic measures: Norms	Non-occupational voice user		Occupational voice user		MW score	*p*-value
		Mean	s.d	Mean	s.d		
Age of patient (years)	-	50.026	13.779	45.161	13.641	487.500	0.223
RSI	< 13 (Abraham & Kahinga, 2022)	24.094	18.671	22.586	10.091	457.500	0.929
VHI	0 ≤ 7 (Caffier et al., 2021)	66.000	39.742	67.759	32.467	443.000	0.927
Jitter (%)	0.591 (Males) 0.590 (Females) (Teixeira et al., 2013)	1.302	1.268	1.655	2.436	295.500	0.747
Shimmer (dB)	0.218 (Males) 0.918 (Females) (Teixeira et al., 2013)	7.150	5.233	6.770	5.710	263.500	0.347
Fundamental frequency (Hz)	120 (Males) 206 (Females) (Teixeira et al., 2013)	202.654	81.094	213.480	61.875	254.000	0.261
Noise to harmonic ratio (dB)	9.632 (Males) 11.04 (Females) (Teixeira et al., 2013)	0.087	0.113	0.092	0.130	283.000	0.573
Maximum Phonation Time (s)	25–35 (Males) 15–25 (Females) (Teixeira et al., 2013)	9.204	6.116	11.576	6.294	249.000	0.155

Note: Raw data obtained from Dr Carl Swanepoel and Giselle Maartens, Pretoria Voice Clinic. Table developed and calculations performed by the authors. Please see the full reference list of the article, Bhaila, Y.M., Van der Linde, J., Milton, C., & Graham, M.A. (2025). Incidence and nature of functional voice disorders. *South African Journal of Communication Disorders, 72*(1), a1117. https://doi.org/10.4102/sajcd.v72i1.1117, for more information.

MW, Mann-Whitney; s.d., standard deviation; RSI, reflux symptom index; VHI, voice handicap index; Hz, hertz; dB, decibel.

Patients were frequently referred to the ENT from other specialist medical practitioners (*n* = 36; 41.9%) and from GMPs (*n* = 23; 26.7%). Small percentage of patients were self-referred (*n* = 8; 9.3%) and (*n* = 2) 2.3% were referred by an SLP. All patients (*N* = 86; 100%) were referred from the ENT specialist to the SLP for voice therapy.

## Discussion

The incidence rate of 16.67% from a population of patients who attended a voice clinic emphasises the importance of recognising and acknowledging FVDs as a notable health consideration within this population. Although not statistically significant, the gender distribution within the sample, with a majority of females, emphasises the importance of recognising and addressing gender-specific differences in the assessment and management of FVDs. Thus, the research findings align with existing literature, reporting a higher prevalence of voice disorders among women (Bainbridge et al., 2017). This was highlighted by a previous study, which found that females were 56.0% more likely to experience a voice disorder than males (Bainbridge et al., 2017). In another study exploring prevalence of voice disorders, gender was identified as a risk factor contributing to ever-present voice disorders, and women were reported to experience voice disorders more often than men (Lyberg-Åhlander et al., 2019).

In this study, the average age of patients experiencing an FVD was 47.26 years, with a lower prevalence among young and middle-aged adults. In contrast, a higher prevalence of voice disorders was found in women and men older than the age of 85 years in a previous study (Lyberg-Åhlander et al., 2019).

The prevalence of LPR as a secondary diagnosis highlights the significance of considering comorbid conditions in the assessment and management of FVDs. Lechien et al. (2017) found that LPR leads to histopathologic changes in the vibratory margin of the vocal folds, leading to changes in the mucosa and thus, a voice disorder. Furthermore, hoarseness was reported as the most common symptom of FVD in this patient population, which could potentially be related to the presence of LPR (Lechien et al., 2017). There is a need for future research to investigate the pathophysiology of hoarseness relating to and the association and potential causality between LPR and voice disorders, as many participants were diagnosed with LPR (39.5%) in this study. Ear, nose, and throat specialists along with SLPs should ensure a comprehensive package of care, which would address both the primary voice disorder and related health issues to provide holistic care.

The wide array of voice-related signs and symptoms reported by patients describe the multifaceted nature of FVDs. The complexity necessitates a comprehensive assessment that accounts for the diverse complaints of affected individuals, ensuring that underlying causes are thoroughly addressed. The signs and symptoms should be explored in future studies to further describe the nature of FVDs and to explore potential confounding factors as various multiple were explored in the study.

The comparison between OVUs and non-OVUs, while not revealing significant differences, highlights the need to reconsider the factors influencing voice-related issues among this population. Although the VHI may not fully capture the occupational demands of professional voice users, it provided a standardised measure across both groups. Future studies could incorporate occupation-specific instruments to capture these nuances.

Furthermore, the highest prevalence of FVDs was found to be in the teaching and singing occupations. This encourages a broader perspective when considering the risk factors associated with FVDs. This study points to a possible gap regarding the relationship between laryngopharyngeal reflux and FVDs; however, further review of existing literature is needed to confirm whether this gap has already been addressed.

This study contributes to the current body of knowledge surrounding voice disorders in the South African context. It highlighted the need for continued research to explore the risk factors within the population, such as occupation and comorbid conditions. More studies exploring the incidence of FVDs are needed among different populations and healthcare settings (i.e., both private and public) to better understand the incidence of FVDs with a larger sample size. Moreover, the therapeutic outcomes must be explored from an SLP and ENT perspective, along with adherence to therapy.

This study is particularly significant as it provides the first comprehensive insight into the incidence and nature of FVDs in a voice clinic in SA. It outlines the need for further research and the development of strategies for prevention, early identification, and treatment. Given the high incidence and prevalence of FVDs, particularly among OVUs, these data support the need for further research in LMICs, where such disorders may be underreported or underdiagnosed. This study lays the foundation for research on the psychological and emotional impact of FVDs, which could contribute to further unpacking the nature of FVDs. Lastly, more research is needed to better understand the course of FVDs, how they develop, how they change over time, and what therapy outcomes are.

### Limitations

The reliance on data from a single interdisciplinary voice clinic may limit the generalisability of findings to a broader population. The study lacks an in-depth exploration of socioeconomic factors that could influence the results as the study was conducted in a private healthcare facility. Acoustic measures such as jitter, shimmer and harmonic-noise ratio may have reduced the validity in patients with severe grades of dysphonia or in cases of single irregularities. Thus, it may not have fully reflected the severity of the voice disorder. A limitation of this study is that auditory-perceptual ratings were performed by a single clinician. As inter-rater reliability was not assessed, we cannot quantify variability across raters. This limits the generalisability of the perceptual findings, and future studies should include multiple independent raters to establish both intra- and inter-rater reliability. Lastly reported lifestyle factors relied on self-reported data, introducing the possibility of social desirability bias.

## Conclusion

This study was the first of its kind in this context, thus filling a critical gap in the available literature on the incidence and nature of FVDs. The use of interdisciplinary data allowed for a comprehensive analysis and provided a perspective on medical and SLP perspectives. Furthermore, the use of both descriptive and inferential statistics provided a detailed and thorough analysis of the available data.

This study provides significant insights into the landscape of FVDs at a private healthcare clinic in SA, highlighting their noteworthy incidence, gender-specific prevalence, and clinical characteristics. The prevalence of MTD and LPR as primary and secondary diagnoses emphasises the clinical complexity of FVDs. The diversity of reported voice-related symptoms and the comparison of OVUs and non-OVUs emphasise the importance of an interdisciplinary approach. These findings lay the foundation for improved prevention and treatment strategies, ultimately enhancing the quality of life for individuals with FVDs in SA and beyond.
